# Lunotriquetral coalition and ulnar impaction syndrome: a pictorial essay

**DOI:** 10.1590/0100-3984.2017.0085

**Published:** 2019

**Authors:** Derik L. Davis

**Affiliations:** 1 Department of Diagnostic Radiology & Nuclear Medicine, University of Maryland School of Medicine, Baltimore, Maryland, USA.

**Keywords:** Lunotriquetral coalition, Ulnar impaction syndrome, Wrist, Coalizão lunatotriquetral, Síndrome da impactação ulnar, Punho

## Abstract

Lunotriquetral coalition and ulnar impaction syndrome are among the spectrum of pathology encountered at the medial wrist. The co-existence of these entities in the same wrist is rare. The purpose of this pictorial essay is to present the etiology, clinical course, imaging findings, and treatment of lunotriquetral coalition and ulnar impaction syndrome, and co-existing disease.

## Abstract (pt)

Coalizão lunatotriquetral e síndrome da impactação ulnar estão incluídas no espectro de doenças do punho medial. A coexistência dessas entidades no mesmo pulso é rara. O objetivo deste ensaio pictórico é apresentar a etiologia, o curso clínico, os achados de imagem e o tratamento da coalizão lunatotriquetral e da síndrome da impactação ulnar, e a coexistência dessas doenças.

## INTRODUCTION

Lunotriquetral coalition and ulnar impaction syndrome (also referred to as ulnocarpal loading or ulnar abutment) are among the spectrum of pathology encountered at the medial wrist. The co-existence of these entities in the same wrist is rare. The purpose of this pictorial essay is to present the etiology, clinical course, imaging findings, and treatment of lunotriquetral coalition, ulnar impaction syndrome, and co-existing disease.

## LUNOTRIQUETRAL COALITION

### Etiology

The carpus of the wrist arises from undifferentiated mesenchyme between the fourth and eighth weeks of fetal life^(^^1^^)^. All carpal bones originate normally from a single cartilaginous anlage. Synovial joint spaces appear between individual carpal bones following an orderly period of cellular apoptosis. The failure of the lunate and triquetrum to separate by the tenth week results in life-long coalition^(^^2^^)^.

Mutations of the NOG gene have been linked to the development of carpal coalition. The NOG gene encodes for the noggin protein which inhibits bone morphogenic proteins. Deficiency in production of the noggin protein has been implicated in the absence of normal cellular apoptosis in carpal coalition^(^^3^^)^.

### Epidemiology, classification and clinical course

**Figure 1:**
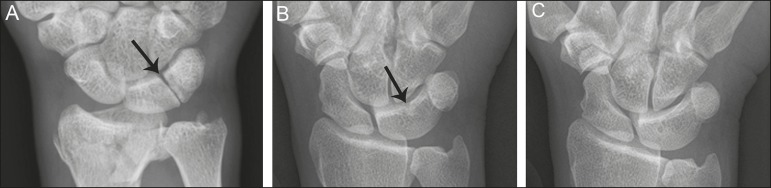
Lunotriquetral coalition Minnaar types 1, 2 and 3. **A:** Type 1 (arrow) is a fibrous syndesmosis between the lunate and triquetrum, in this case found incidentally in a patient presenting with acute distal radius and ulna fractures. **B:** Type 2 is incomplete bony coalition, as evidenced by a small residual lucent cleft in this case (arrow). **C:** Type 3 is complete bony coalition.

**Figure 2:**
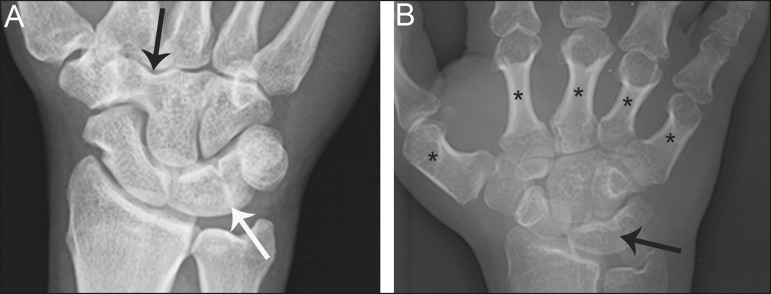
Different examples of Minnaar type 4 lunotriquetral coalition. **A:** Lunotriquetral coalition (white arrow) accompanying by capitatotrapezoid coalition (black arrow). **B:** Multiple short metacarpal bones (asterisks) associated with lunotriquetral coalition.

Carpal coalition is a rare developmental anomaly, and the incidence is 0.1% and 1.6% in white and black populations, respectively^(^^1^^)^. Lunotriquetral coalition is by far the most common form of carpal coalition, representing nearly 90% of all cases^(^^1^^,^^4^^)^. Lunotriquetral coalition is classified by the degree of bony synostosis between the lunate and triquetrum (Figures 1 and 2). The Minnaar classification system divides lunotriquetral coalition into four categories: type 1 coalition is a pseuodarthrosis, without bony fusion; type 2 coalition has partial bony fusion; type 3 has complete bony fusion; and type 4 demonstrates complete bony fusion in association with other carpal bone anomalies^(^^5^^)^.

Typically, all forms of lunotriquetral coalition are asymptomatic and represent incidental findings. Rarely, cases of type 1 coalition have been described as a cause of medial wrist pain, stemming from degenerative arthritis, chronic repetitive microtrauma or acute disruption of the pseudoarthrosis^(^^2^^,^^4^^)^. The bony synostoses present in types 2 through 4 are theorized to prevent motion at the lunotriquetral articulation, and thus prevent symptoms. If conservative therapy fails to provide pain relief for symptomatic type 1 coalition, surgical management with arthrodesis is an effective treatment^(^^2^^,^^4^^)^.

## ULNAR IMPACTION SYNDROME

### Etiology

**Figure 3:**
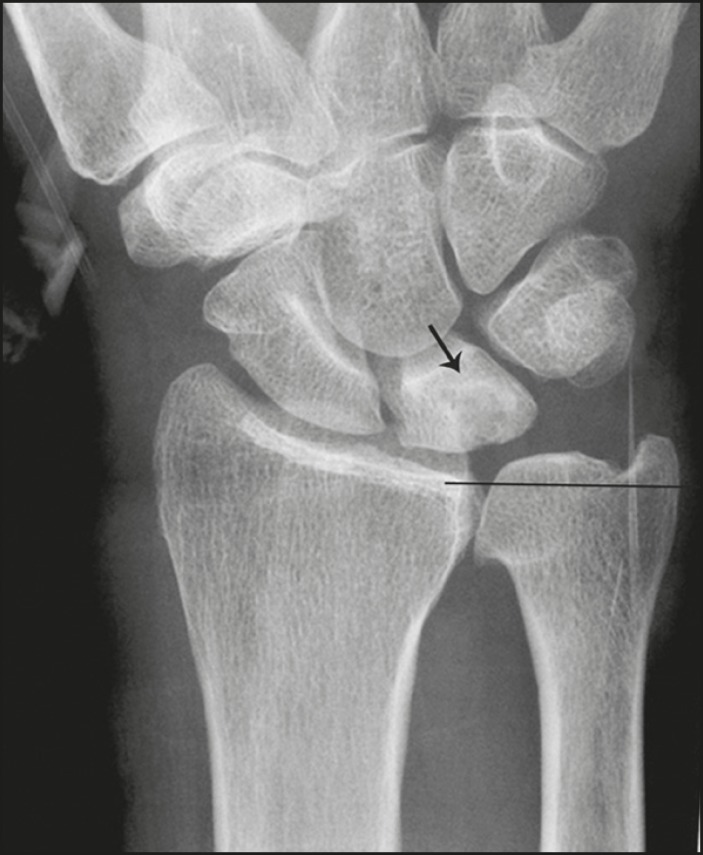
Positive ulnar variance. The ulnar head is distal to the articular surface of the radius (line). Prominent subchondral cystic changes (arrow) are associated with the ulnocarpal joint surface of the lunate in this patient.

**Figure 4:**
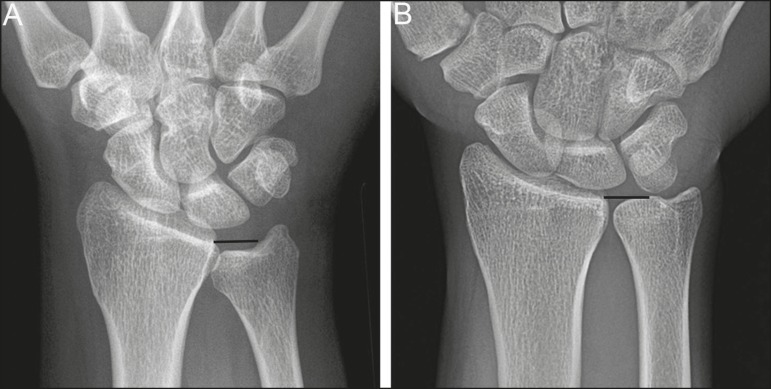
Negative and neutral ulnar variance. **A:** In negative ulnar variance, the ulnar head is proximal relative to the articular surface of the radius (line). **B:** In neutral ulnar variance, the radius and ulna are equal length (line).

Chronic repetitive bony loading between the ulna and medial bones of the proximal carpal row is the underlying cause of ulnar impaction syndrome, and ulnar variance is a key risk factor^(^^6^^-^^9^^)^. Positive ulnar variance is the most commonly associated predisposing factor, although ulnar impaction syndrome also occurs in the setting of neutral or negative ulnar variance (Figures 3 and 4). Positive ulnar variance is defined by the presence of the ulnar head distal to the articular surface of the radius at the distal radioulnar joint on a posteroanterior radiograph of the wrist with the forearm in neutral rotation. Negative ulnar variance represents a proximal position of the ulnar head relative to the radial articular surface, while an equal length of both bones defines neutral ulnar variance. Positive ulnar variance may be idiopathic or acquired. Surgical resection, premature growth plate closure and fracture malunion of the distal radius are commonly encountered acquired etiologies of positive ulnar variance^(^^6^^-^^9^^)^.

### Epidemiology and clinical course

Ulnar impaction syndrome is a common cause of medial-sided wrist pain, although the abnormality is likely under reported due to frequent non-recognition of the diagnosis^(^^7^^)^. Symptomatic patients present with wrist swelling and functional limitations at the radiocarpal and distal radioulnar joints, in addition to pain^(^^7^^,^^8^^)^. The typical clinical course manifests as chronic ongoing bouts of symptoms which are aggravated by activity and wane with rest^(^^6^^)^. Ulnar impaction syndrome is known to occur from repetitive microtrauma in connection with participation in racket sports and gymnastics, although non-sports-related causes such as long-term computer keyboard use also has been described^(^^8^^,^^9^^)^.

Ulnar impaction syndrome is associated with a spectrum of pathology at the medial wrist, including triangular fibrocartilage complex, cartilage, bone and joint injury. The Palmer classification system is a well-known scheme for categorizing acute and chronic triangular fibrocartilage complex, bone and joint abnormalities at the medial wrist^(^^9^^)^. Radiographic findings include subchondral sclerosis and subchondral cystic changes in the ulnar head, lunate and/or triquetrum. Late radiographic findings show advanced osteoarthritis at the medial wrist, although early pathologic changes may be radiographically occult^(^^6^^)^. Magnetic resonance imaging is highly sensitive to detect subchondral bone marrow edema in the early stages of ulnar impaction syndrome, and is also able to characterize fully triangular fibrocartilage complex tears and associated osteochondral injuries^(^^9^^)^.

Operative management is useful for treatment of ulnar impaction syndrome in patients who fail conservative therapy. Surgical options include ulnar shortening osteotomy, distal ulnar resection or distal radioulnar arthrodesis with distal ulnar pseudoarthrosis^(^^8^^,^^10^^)^.

## CO-EXISTENT LUNOTRIQUETRAL COALITION AND ULNAR IMPACTION SYNDROME

The presence of lunotriquetral coalition and ulnar impaction syndrome in the same wrist is rare. The rarity of this combination stems from the fact that lunotriquetral coalition is only present in less than 2% of the population^(^^1^^)^, although the true incidence of co-existent disease with ulnar impaction syndrome is unknown. To the author's knowledge, no prior case of congenital lunotriquetral coalition has been reported in combination with ulnar impaction syndrome in the literature. However, one publication in the literature has reported five cases of ulnar impaction syndrome as a complication of lunotriquetral arthrodesis following operative management for lunotriquetral injury^(^^11^^)^. The acquired form of lunotriquetral fusion following surgical arthrodesis is functionally analogous to congenital lunotriquetral coalition in the author's opinion.

**Figure 5:**
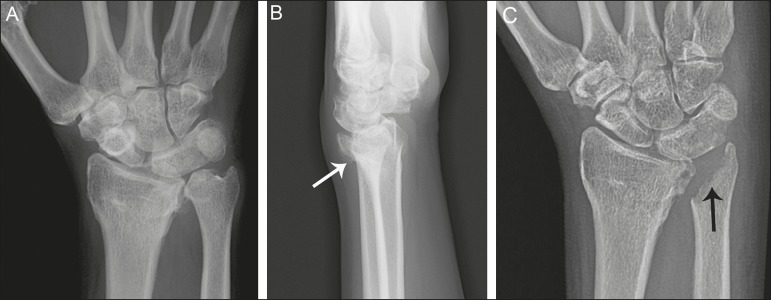
Symptomatic type 2 lunotriquetral coalition complicated by ulnar impaction syndrome requiring surgical management. **A,B:** Anteroposterior and lateral radiographs show acquired positive ulnar variance as the long term sequela of distal radius fracture malunion, with persistent chronic loss of radial length and dorsal tilt (arrow) following healing. **C:** Surgical resection of the distal ulna (arrow) was performed for operative management.

**Figure 6:**
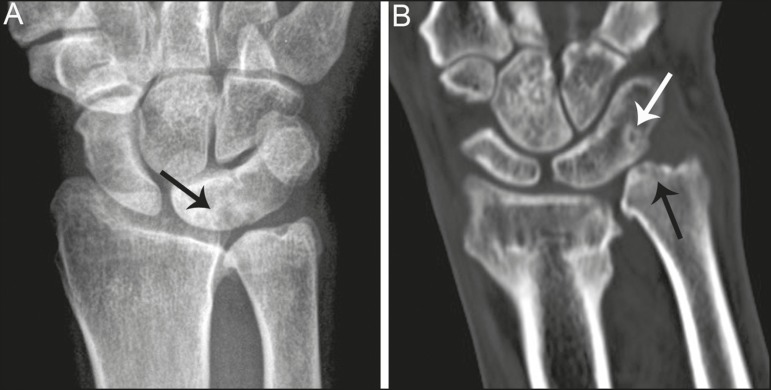
Locations of bony pathology in ulnar impaction syndrome complicating lunotriquetral coalition. **A:** Posteroanterior radiograph shows positive ulnar variance, type 2 lunotriquetral coalition, and subchondral cysts at the ulnocarpal articular surface of the lunate (arrow). **B:** Coronal image of unenhanced computed tomography shows positive ulnar variance, type 3 lunotriquetral coalition, and subchondral cysts in the ulnar head (black arrow) and triquetrum (white arrow).

**Figure 7:**
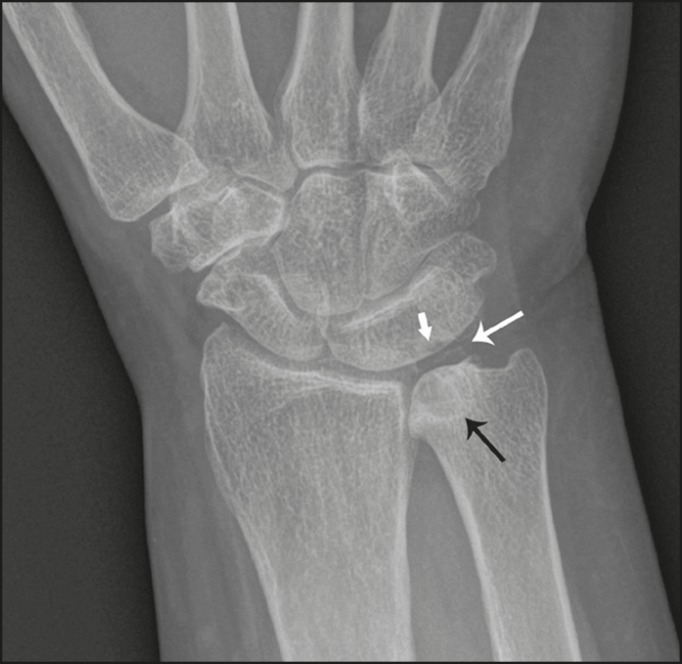
Isolated type 3 lunotriquetral coalition complicated by ulnar impaction syndrome. Subtle sclerosis and subchondral cyst formation (short white arrow) is present in a central location along the ulnocarpal surface of the lunotriquetral coalition, in addition to more prominent cystic changes and central osteophyte formation at the ulnar head (black arrow). Linear chondrocalcinosis is centered in the ulnocarpal joint space (long white arrow).

**Figure 8:**
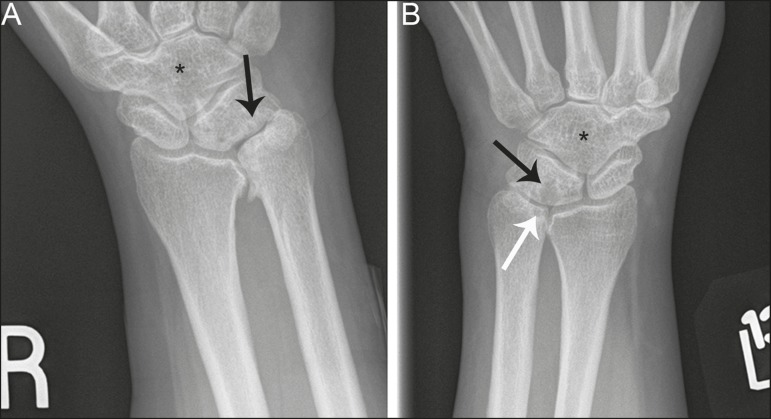
Syndrome-related lunotriquetral coalition complicated by ulnar impaction syndrome. **A,B:** Right and left anteroposterior radiographs of the wrists show type 4 lunotriquetral coalition with associated bony coalition of the entire distal carpal row bilaterally (asterisks). Subchondral cystic changes are present in the left ulnar head (white arrow) and the ulnocarpal aspect of the bilateral lunotriquetral coalitions (black arrows).

**Figure 9:**
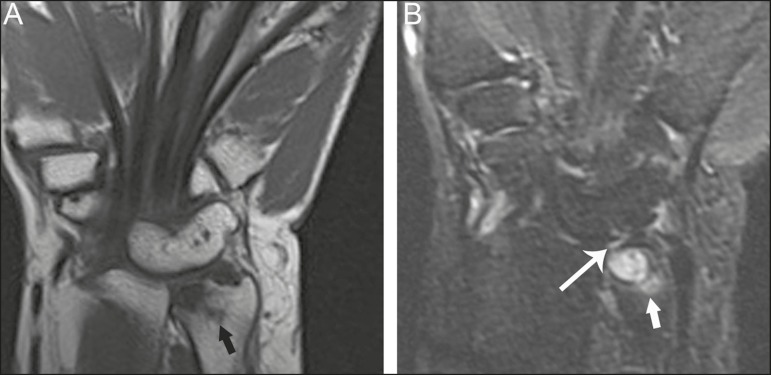
Type 3 lunotriquetral coalition complicated by ulnar impaction syndrome on magnetic resonance imaging. **A,B:** Coronal T1-weighted and coronal STIR-weighted magnetic resonance images show bone marrow edema (short arrows) adjacent to a large subchondral cyst at the ulnar head, and a triangular fibrocartilage tear at the radial attachment (long arrow).

Typically, only isolated type 1 lunotriquetral coalition is known to be symptomatic^(^^2^^,^^4^^)^. In the author's experience, the other types of lunotriquetral coaliation may be associated with medial-sided wrist symptoms when co-existent ulnar impaction syndrome is present (Figure 5). This is analogous to symptoms developing from ulnar impaction syndrome following surgical lunotriquetral arthrodesis^(^^11^^)^. For lunotriquetral coalition in combination with ulnar impaction syndrome, signs of osteochondral pathology may manifests at the ulnar head, lunate and/or triquetrum (Figure 6). Ulnar impaction syndrome may occur in association with the isolated form of lunotriquetral coalition or as part of a syndrome (Figures 7 and 8). Magnetic resonance imaging is sensitive for full characterization of discrete lesions found in association with both lunotriquetral coalition and ulnar impaction syndrome (Figure 9)^(^^9^^)^.

Surgical treatment for patients with medial-sided wrist symptoms who fail conservative management in the presence of lunotriquetral coalition and ulnar impaction syndrome should address the underlying cause. Options for management of ulnar impaction syndrome would include ulnar shortening osteotomy, distal ulnar resection or distal radioulnar arthrodesis with distal ulnar pseudoarthrosis^(^^8^^,^^10^^)^, while lunotriquetral arthrodesis would be the typical option for symptomatic type 1 coalition^(^^2^^,^^4^^)^.

## CONCLUSION

Lunotriquetral coalition and ulnar impaction syndrome are among the spectrum of medial wrist pathology. Knowledge of the etiology, clinical course, imaging findings, and treatment of lunotriquetral coalition, ulnar impaction syndrome, and co-existent disease will aid in the care of patients who present with medial wrist symptoms.
